# Knowledge about birth preparedness and complication readiness and associated factors among primigravida women in Addis Ababa governmental health facilities, Addis Ababa, Ethiopia, 2015

**DOI:** 10.1186/s12978-020-0861-z

**Published:** 2020-01-29

**Authors:** Ayelech Kidanemariam Mulugeta, Berhanu Wordofa Giru, Balcha Berhanu, Tefera Mulugeta Demelew

**Affiliations:** 1 Kotebe Health Center, Addis Ababa Health Bureau, Addis Ababa, Ethiopia; 2 School of Nursing and Midwifery, College of Health Sciences, Addis Ababa university, PO. Box 4412, Addis Ababa, Ethiopia

**Keywords:** Birth preparedness, Complication readiness, Primigravida, Knowledge, Health facilities

## Abstract

**Background:**

Globally, every minute, at least one woman dies from complications related to pregnancy or childbirth. The situation is more serious for women in Sub-Saharan Africa which also include Ethiopia. Birth preparedness is a strategy to promote the timely use of skilled maternal and neonatal care, especially during childbirth. Based on the theory, preparing for childbirth reduces delays in obtaining this care. In adequate preparation for rapid action in the event of obstetric complications are well documented factors contributing to delay in receiving skilled obstetric care. Hence, the aim of this study was to assess the knowledge of birth preparedness and complication readiness and its associated factors among primigravida in Addis Ababa Governmental Health Facilities.

**Methods:**

A quantitative facility based cross-sectional study design and client exit interview questionnaire were used. Simple random and census sampling was used to select the health care facilities and study participants, accordingly. The data were entered using Epidata version3.1 and analysed by window statistical package for social science version 20 software. Logistic regression model was used to assess the knowledge by predictor’s variables.

**Result:**

From 442 respondents the response rate was 422 (95.5%). Based on finding, the respondents were knowledgeable on danger signs in pregnancy, labour, postnatal and new born neonate 113(26.8%), 47(11.1%), 60(14.2%) and 46(10.9%), respectively. According to birth preparedness, 64 (15.2%) of primigravida women were knowledgeable. In this study, factors associated with knowledge of birth preparedness and complication readiness were found to be being married [AOR = 0.110, 95%CI (0.026, 0.461);], house hold monthly income of 1000–3000 [AOR = 3.362(1.203,9.393);], knowledgeable for key danger signs of labour with [AOR = 3.685, 95%CI (1.157, 11.737);] and knowledgeable for key danger signs of post-partum period with [AOR =5.117, 95%CI (1.388, 18.863);].

**Conclusion:**

The knowledge of primigravida women for birth preparedness and its complication readiness was low. Information given about danger sign and birth preparedness during ANC follow up was not comprehensive. Therefore, family health care providers, health facility, other partners, program level managers and policy makers take their responsibility and work together to improve the health education service and increase knowledge on birth preparedness and complication readiness through easily accessible health education strategies.

## Plain English summary

Birth preparedness and complication readiness is a strategy that has been globally endorsed as an essential component of safe motherhood programs to reduce delays for care to promote the timely use of skilled maternal and neonatal care.

Client exit interview of 442 primigravida (first pregnancy) women in Antenatal care clinic in health centres and hospitals was revealed, 15.2% of primigravida women were knowledgeable about birth preparedness with being married, house hold monthly income of 1000–3000, knowledgeable for key danger signs of labour, and knowledgeable for key danger signs of post-partum period associated with the outcome variable.

Family health care providers, health facility, other partners, program level managers and policy makers take their responsibility and work together to improve the service and increase knowledge on birth preparedness and complication readiness.

## Background

Although women play a major role in the rearing of children and the management of family affairs, their loss from maternity-related causes is a significant social and personal tragedy [1]. As estimated by the World Health Organization (WHO), about 580,000 women die each year from complications arising from pregnancy and childbirth [1–3] every minute, at least one woman dies from complications related to pregnancy or childbirth. The situation is more serious for women in Sub-Saharan Africa where one in every 16 women dies because of pregnancy related causes. In fact, Sub Saharan Africa incurs 98% of maternal deaths [4]. Three-quarters of the 4 million global neonatal deaths occur in the first week of life and stillbirth rate is 32 per 1000 deliveries, of which 24–37% occur during the intrapartum period [5].

Birth preparedness and complication readiness (BP and CR) is a strategy that has been globally endorsed as an essential component of safe motherhood programs to reduce delays for care to promote the timely use of skilled maternal and neonatal care, especially during childbirth, based on the theory that preparing for childbirth and being ready for complications reduces delays in obtaining this care [6–8]. Moreover, it helps to ensure that women can reach professional delivery care when labour begins. In addition, complication readiness can help to reduce the delays that occur when women experience obstetric complications, such as recognizing the complication and deciding to seek care, reaching a facility where skilled care is available and receiving care from qualified providers at the facility [7].

BP and CR and safe motherhood program approaches; Thaddeus and Maine (1994) have provided the safe motherhood community with an explanatory model of maternal mortality that identifies delays in seeking, reaching and obtaining care as the key factors leading to maternal death. This explanatory model, known as the Three Delays Model categorizes delays into three types: delays in seeking care, delays in reaching care, and delays in receiving adequate care once at the point of service. This strategy promotes the timely use of skilled maternal and neonatal care, especially during childbirth, based on the theory that preparing for childbirth reduces delays in obtaining care. A birth plan/emergency preparedness plan include identification of following elements: knowledge of danger signs, the desired place of birth, the preferred birth attendant, the location of the closest appropriate care facility, funds for birth-related and emergency expenses, a birth companion, support in looking after the home and children while the woman is away, transport to a health facility for the birth, transport in the case of an obstetric emergency, and identification of compatible blood donors in case of emergency [6–10].

In many societies in the world, cultural beliefs and lack of awareness inhibit preparation in advance for delivery and expected baby. Since no action is taken prior to the delivery, the family tries to act only when labour begins. The majority of pregnant women and their families do not know how to recognize the danger signs of complications. When complications occur, the unprepared family will waste a great deal of time in recognizing the problem, getting organized, getting money, finding transport and reaching to the appropriate referral facility [11].

In Ethiopia, an estimated 2.9 million women give birth every year. Of these, approximately 25,000 women and girls die each year and more than 500,000 suffer from complications including obstetric fistula [1, 5]. The levels of maternal mortality and morbidity in the country was 676 per 100, 000 live births accounting for 21% of all death [3, 5]. Of the causes, 6% were attributable to complications from abortion [12].

Some studies in Ethiopia showed only 22% & 16.5% of respondents were prepared for birth & its complication respectively [3, 13]. And factors affecting it were role of husbands, level of education, parity and absence of community-based support services, monthly income, ANC visit & knowledge of obstetric complications [3, 13, 14]. However, there is limitation of evidences selectively on primigravida women who have no any experience on BP &CR in our country. Therefore, this study entitled to determine knowledge of BP & CR and its associated factors among primigravida women in Addis Ababa Governmental health facilities.

## Methods

### The study area and period

This research was conducted in Addis Ababa city governmental Health care facilities providing ANC service during the data collection period. Addis Ababa has ten sub cities and 116 woreda [15]. The city had 14 public hospitals, of which eight were managed by the FMOH while the remaining six hospitals and additional 84 health centres were owned by the City Administrative Health bureau. The reason for selecting Addis Ababa government health centres and hospitals were low/free of charge for ANC service. Hence it was convenience for high flow of ANC attendance. The study was conducted in randomly selected health centres and hospitals of Addis Ababa which had been providing antenatal care from April1 20—May 20, 2015.

### Study design

A facility based cross-sectional quantitative study design was conducted.

### Source population

All primigravida women, who came to attend ANC from all government health facilities during data collection period.

### Study population

Primigravida women attending ANC service at selected health facilities (Hospitals and Health centres) during data collection period.

### Eligibility criteria

#### Inclusion criteria

All primigravida women, who were attending ANC follow up during the study period

#### Exclusion criteria

Primigravida women who were critically or mentally ill at the time of interview.

### Sample size determination

The sample size was estimated using a single population proportion formula. Since there were no previous studies done in that area which can estimate the problems specifically in primigravida, a prevalence level that can estimate maximum sample size (50%), marginal error (d) 0.05, with 95% confidence interval certainty and alpha error 0.05 were considered. Based on these assumptions, a total sample size was 384, with 15% non-response rate, the total sample size was = **442**.

### Sampling procedures

Simple random sampling using lottery method was used to select five health centres and two hospitals. The total sample size was proportionally allocated for the five health centers and two hospitals (Gandhi and Yekatite 12), depending on the daily average client flow in each health facilities. Considering 6-months performance in 22 working days of each month, the daily average client flow of those selected Health Centres was Twenty-Five while it was seventy-five & thirty-five at Gandhi & Yekatit 12 hospitals, respectively. Therefore, the study population was 550 for each health centres, while 1650 and 770 at two hospitals accordingly. Then, the final sample size was proportionally allocated for each facility as 47 for each health centres, and 141 and 770 was to Ghandi and Yekatit 12 hospitals. All primigravida women fulfilling the inclusion criteria were included as Individual study participants.

### Data collection procedure

Structured questionnaire adapted from JHPIEGO BP monitoring tool, and reviewed literature on monitoring BP/CR and awareness of danger signs in Mekele town (7, 3, 12). These questionnaires were modified to include all relevant variables to meet the objectives and considering the study area and setup. The questionnaire had two sections and three parts. In section one, two parts were included, which are participants sociodemographic characteristics (age, marital status, educational level, income, family size….) and obstetrics characteristics (such as: pregnancy stage at first visit of ANC, frequency of ANC, and gestational age) was included. Under section two, 12 questions were included to assess participants knowledge on birth preparedness and complication readiness (such as pregnancy, labor, post-partum and new born neonate related questions). Besides, questions designed to asses participants’ awareness to assess birth preparedness were also included. English version was translated to Amharic for better understanding of the enumerators and respondents. Back translation from Amharic to English to check its original meaning**.**

### Study variable

#### Dependent variables

Knowledge of birth preparedness and complication readiness

#### Independent variables

- Socio demographic characteristics (Age, marital status, occupation, income and maternal education), Obstetric factors (Time of ANC visit, No. of ANC visits and Gestational age), Awareness of danger signs, Husband’s factors (occupation, education and income), House hold income

### Operational definition/measuring scale

The operational definition or measuring scale adapted from JHPIEGO monitoring birth preparedness and complication readiness [7].

- **Knowledgeable about birth preparedness and its complication**: A woman was considered knowledgeable if she could spontaneously mention all four components: - skilled providers, saved money, identified place of delivery and identified mode of transport.
- **Knowledgeable of key danger signs:** If a woman spontaneously mentioned the expected key danger signs of each period as follows
  - **during pregnancy**: all three key danger signs for pregnancy (Severe vaginal bleeding**,** swollen hands/ face and blurred vision).
  - **labour/childbirth**: four all key danger signs for Labour/childbirth which were: Severe vaginal bleeding**,** Prolonged labour (> 12 h)**,** convulsions and retained placenta.
  - **postpartum:** three all key danger signs for postpartum which were (Severe vaginal bleeding, foul smelling vaginal discharge and high fever).
  - **of the new born**: four all key danger signs of new born neonate which include: **(**Convulsions/spasms/ rigidity, difficult/fast breathing, very small baby and lethargy/unconsciousness).
- **Primigravida women:** refer to the women who were pregnant for the first time.

### Data quality measurement

To keep the quality of the data a standard questionnaire was adapted, developed and 5% pre-test was done for accuracy and consistency on primigravida women outside the study facilities. The questionnaire was tested for the relevance of dependent and independent variables to avoid any confusion during actual data collection period. The principal investigator and some data collectors were checked 22 antenatal care attendants (5%) of primigravida women response 1 week prior to the actual data collection period outside the study facilities, in Yeka sub-city, wereda 11 health center. This was helpful for the investigator to screen out vague questions and some of the question item was modified. In data collection seven data collectors (diploma in nursing) and three supervisors (BSc in nursing) were participated. The selected data collectors were familiarized with the questionnaire by obtaining one-day orientation on the objective of the study and the content of the instrument. The principal investigator followed and supervised the enumerators throughout the data collection period. During data collection process each questionnaire was checked daily by the supervisor and principal investigator for its completeness.

### Data analysis procedures (data entry and analysis)

Data was entered and coded into a computer using Epi Data version 3.1 then exported to SPSS version 20 and analysed. Descriptive statistics with frequency, percentages, tables, graphs and cross-tabulations were used. In addition, logistic regression statistical models, using bivariate and multivariable logistic regression analysis method were used for analysis. Covariates having significant association (*P* < 0.05) with knowledge of BP and CR in bivariate analysis were entered to multivariable analysis to reduce confounding factors and identify predictor variables. Confidence interval of 95% to see the precision of the study and the statistical association was considered as significant if *p*-value was less than 0.05 and logistic regression tables were also used to present the data.

## Results

### Socio -demographic characteristics of the participants

This study was conducted on a total of 442 primigravida women those who came for ANC service in health centres and hospitals. Out of these 422 primigravida women, the majority 347 (82.6%) were between the age of 21 and 32. Of these 422 (100%) participants, the majority 359 (85.1%) were married. 287 (68%) participants were followers of Orthodox Christian religion. In addition to this, almost half 187 (44.3%) of the participants were from Amhara ethnic group. Over one third respondents 151 (35.8%) were house wives and also more than half of the participants 247 (58.5%) had secondary education and above. The rest 60 (14.2%) were not literate.

Concerning the socio-economic background of the participants (*N* = 422), the study showed that 182 (44.5%) had a monthly income between 1000.00–3000.00 birr. There was almost similar figure 90 (21.3%) and 92 (21.8%) had no any monthly income and less than 1000.00-birr income, respectively. On the other hand, the majority 294 (79.9%) of the participant’s husband have secondary education and above. Majority of the husbands 153 (36.3%) and 120 (28.4%) employed in the private and government sector, respectively. Almost half of the participant’s husbands earn monthly income between 1000.00–3000.00 birr. More than half of the participants 231 (54.9%) had one or two-family members. As a cumulative, half of the participants 212 (50.2%) had more than 3000.00 birr of household monthly income (Table 1).

**Table 1:** Distribution of socio-demographic variables of respondents in Addis Ababa Government Health facilities, (*n* = 422) May, 2015

Variable	Frequency	Percent
Age in years		
15–20	46	10.9
21–26	189	44.8
27–32	158	37.8
33+	29	6.9
Total	422	100
Marital status		
Married	359	85.1
Single	44	10.4
Widowed	14	3.3
Divorced	5	1.2
Total	422	100
Religion		
Orthodox	287	68.0
Protestant	81	19.2
Muslim	52	12.3
(Others)	2	.5
Total	422	100
Ethnicity		
Amhara	187	44.3
Oromo	100	23.7
Gurage	58	13.7
Tigre	51	12.1
Siltie	16	3.8
(Others)^a^	10	2.4
Total	422	100
Occupation		
Housewife	151	35.8
Gov.employee	101	23.9
Pvt. employee	109	25.8
Pvt. business	53	12.6
(Others)^b^	8	1.9
Total	422	100
Educational status		
Not literate	60	14.2
Primary	115	27.3
Secondary and above	247	58.5
Total	422	100
Her monthly Income		
< 1000 birr	90	21.3
1000–3000 birr	182	44.5
> 3000 birr	52	12.3
None	92	21.8
Total	422	100
Husband educational status		
Not literate	18	4.9
Primary	56	15.2
Secondary and above	294	79.9
Not having husband	54	12.8
Total	422	100
Husband occupation		
Government employee	120	28.4
Private employee	153	36.3
Business man	90	21.3
(Other)^c^	5	1.2
Not having husband	54	12.8
Total	422	100
Husband income		
1000 birr	78	21.1
1000–3000 birr	175	47.6
> 3000 birr	115	31.3
Not having husband	54	12.8
Total	422	100
Family size		
1–2	231	54.9
3–4	164	38.9
> 4	27	6.4
Total	422	100
House hold income		
1000 birr	54	12.8
1000–3000 birr	156	37.0
> 3000 birr	212	50.2
Total	422	100

^a^wolayita

^b^Daily labourer

^c^Daily labourer

### Obstetric characteristics of the respondents

A total of 286 (67.8%) primigravida women had receive the first ANC (1 to 4 months). Among all the primigravida women, 175 (41.5%) had more than 7-month gestational age. Out of 422 respondents, 248 (58.8%) of the participants had 2 to 3 times ANC follow up (Table 2).

**Table 2:** Obstetric characteristics of the respondents, in Addis Ababa Government health facilities, (*n* = 422) May, 2015

Variable	Frequency	Percent
First received ANC (in month)		
1–4	286	67.8
5–7	118	28
> 7	18	4.2
Total	422	100
Gestational age (in month)		
1–4	88	20.9
5–7	159	37.7
> 7	175	41.4
Total	422	100
Number of ANC follow up		
1	102	24.2
2–4	248	58.8
> 4	72	17.0
Total	422	100

### Knowledge of danger signs during pregnancy

According to the awareness found from participants primigravida women, great majority of all the participants (355 (84.1%)) knew about serious health problem/s that can occur during pregnancy. The rest of the participants don’t know those signs. Out of those who had awareness; majority of them mentioned vaginal bleeding as danger sign of pregnancy 319 (75.6%). In addition, 288 (61.1%) mentioned severe headache and half of them 211 (50%) reported blurred vision as danger sign of pregnancy, 216 (51.2%) mentioned swollen hands and face. On the other hand, 74(17.5%), 89 (21.1%) and 81 (19.2%) of the participants reported abdominal pain, high fever and reduced foetal movement respectively (Table 3).

**Table 3:** Knowledge about danger signs of pregnancy among primigravida women in A. A. Government Health facilities, May, 2015

Variables	Response	Frequency	Percent
Know any /some serious health problem/s that can occur during pregnancy that could endanger the life of pregnant women (*n* = 422)	Yes	355	84.1
	No	36	8.5
	Don’t know	31	7.3
Mention vaginal bleeding as danger sing during pregnancy (*n* = 355)	Yes	319	75.6
	No	36	8.5
Mention severe head ace as danger sing during pregnancy (*n* = 355)	Yes	288	61.1
	No	97	23.0
Mention blurred vision as danger sing during pregnancy (*n* = 355)	Yes	211	50.0
	No	144	34.1
Mention severe abdominal pain as danger sing during pregnancy (*n* = 355)	Yes	74	17.5
	No	281	66.6
Mention swollen hands and face as danger sign during pregnancy (*n* = 355)	Yes	216	51.2
	No	139	32.9
			84.1
Mention fever as danger sign during (*n* = 355)	Yes	89	21.1
	No	266	63.0
			84.1
Mention Foetus movement reduced or absence and Excessive danger sign during pregnancy (*n* = 355)	Yes	81	19.2
	No	274	64.9
			84.1

### Knowledge of danger signs during labour/childbirth

With regard to serious health problem/s that can occur during labour and child birth that could endanger the life of pregnant women, more than three-fourths of them, and 302 (71.6%) responded had awareness. Out of these 302 primigravida women; 278 (65.9%) mentioned vaginal bleeding and also half of them, mentioned severe headache as danger sign. The respondents who have mentioned convulsion as danger sign were 128 (30.3%) while those who have state fever were 81(19.2%). On the other hand, 53 (12.6%) of the respondents have revealed loss of consciousness as danger sign. Among the respondents 144 (34.2%) expressed labour lasting more than 12 h as danger sign. Out of those 302 primigravida women respondents 82 (19.4%) have mentioned placenta not delivered 30 min after the neonate as their danger sign. There were 5 (1.2%) respondents who have cited fluid coming out of their vagina as danger sign (Table 4).

**Table 4:** Knowledge about danger signs of labour/childbirth among primigravida women in A. A. Government Health facilities, May, 2015

Variables	Response	Frequency	Percent
Any/ some serious health problem/s that can occur during labour and child birth that could endanger the life of pregnant women (*n* = 422)	Yes	302	71.6
	No	65	15.4
	Don’t know	55	13.0
Mention vaginal bleeding as danger sing during labour and delivery (*n* = 302)	Yes	278	65.9
	No	24	5.7
Mention severe head ache as danger sing during Labour and delivery (*n* = 302)	Yes	152	36.00
	No	150	35.00
Mention convulsion as danger sing during Labour and delivery (*n* = 302)	Yes	128	30.3
	No	174	41.2
Mention fever as danger sign during Labour and delivery (*n* = 302)	Yes	81	19.2
	No	221	52.4
Mention Loss of consciousness as danger sign during Labour and delivery (*n* = 302)	Yes	53	12.6
	No	249	59.00
Mention Labour lasting > 12 h (*n* = 302)	Yes	144	34.2
	No	158	37.4
Mention placenta not delivered 30 min after the neonate as danger sign during Labour and delivery (*n* = 302)	Yes	82	19.4
	No	220	52.1
(Others)^a^(*n* = 302)	Yes	5	1.2
	No	297	70.4

Mentioned more than one response

^a^Rapture of amniotic fluid, back ache

### Knowledge of danger signs during post-partum

Out of 422 respondents, 266 (63.0%) stated that they knew the information about danger sign during postpartum period. From those who had the knowledge; 234(55.5%) reported vaginal bleeding and 155 (36.7%) sever head ache as a danger sign. There were also 119 (28.2%) of the respondents who mentioned blurred vision as danger sign during post-partum period. Out of the 266 respondents, who knew about danger sign during post-partum period, 78 (18.5%) have reported convulsion as danger sign (Table 5).

**Table 5:** Knowledge about danger signs of first 42 days after birth among primigravida women in A. A. Government Health facilities, May, 2015

Variables	Response	Frequency	Percent
Danger sign during the first 42 days after birth that could endanger the life of pregnant women (*n* = 422)	Yes	266	63.0
	No	71	16.8
	Don’t know	85	20.1
Mention heavy vaginal bleeding as danger sing during postnatal period (*n* = 266)	Yes	234	55.5
	No	32	7.5
Mention severe head ache as danger sing during postnatal Period (*n* = 266)	Yes	155	36.7
	No	111	26.3
Mention Blurred vision as danger sing during postnatal (*n* = 266) Period (*n* = 266)	Yes	119	28.2
	No	147	34.8
Mention convulsion as danger sing during postnatal period (*n* = 266)	Yes	78	18.5
	No	188	44.5
Mention oedema as danger sing during postnatal period (*n* = 266)	Yes	92	21.8
	No	174	41.2
Mention fever as danger sign during postnatal period (*n* = 266)	Yes	89	21.1
	No	177	41.9
Mention fainting as danger sign during postnatal period (*n* = 266)	Yes	42	10.0
	No	224	53.0
Mention breathing problem as danger sign during postnatal Period (*n* = 266)	Yes	52	12.3
	No	214	50.7
Mention weakness as danger sign during postnatal period (*n* = 266)	Yes	57	13.5
	No	209	49.5
Mention foul smelling vaginal discharge as danger sign during postnatal period (*n* = 266)	Yes	132	31.3
	No	134	31.8
(Others)^a^ (*n* = 266)	Yes	6	2.3
	No	260	60.7

Mentioned more than one response

^a^Loss of appetite, abdominal cramp

### Knowledge about danger signs of new born neonate

From all 422 respondents, 277 (65.6%) stated that they are aware of the information about danger signs of new born neonate. And, these respondents mentioned danger signs of new born neonate; as convulsion (96, 22.7%) very small baby (116, 39.3%)) lethargy or loss of consciousness (98, 23.2%), and difficulty of breathing were (185,43.8%) (Table 6).

**Table 6:** Knowledge about danger signs of new born neonate among primigravida women in A. A. Government Health facilities, May, 2015

Variable	Response	Frequency	Percent
Danger sign of new born neonate birth that could endanger the life of neonate (*n* = 422)	Yes	277	65.6
	No	70	16.6
	Don’t know	75	17.8
Convulsions/spasms/rigidity (*n* = 277)	Yes	96	22.7
	No	181	42.9
Very small baby (*n* = 277)	Yes	166	39.3
	No	111	26.3
Lethargy/unconsciousness (*n* = 277)	Yes	98	23.2
	No	179	42.4
Difficult/fast breathing (*n* = 277)	Yes	185	43.8
	No	92	21.8
(Others)^a^ (*n* = 277)	Yes	19	4.5
	No	258	61.1

Mentioned more than one response

^a^crying and hungry

### Source of information about birth preparedness

**Fig. 1:**
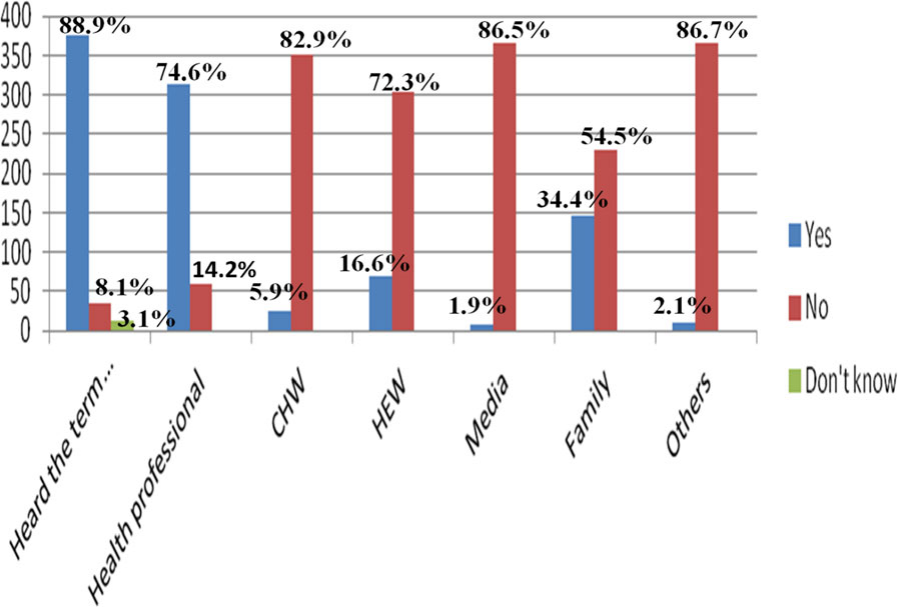
Source of information about birth preparedness, in A. A. Government health facilities (*n* = 422), May, 2015. *Others; friend, neighbours

Out of 422 respondents, 375 (88.9%) have ever heard the word birth preparedness, the rest respondents 34 (8.1%) have never heard while 13 (3.1%) stated as they don’t know about it. Out of 375 respondents who heard about the word birth preparedness, 315 (74.6%) have reported the source of information was health professionals, while the remaining respondents mentioned CHW (Community Health Worker) (25, 5.9%) HEW (Health Extension Workers) (70, 16.6%), media (8,1.9%) family (145, 34.4%), and friends and neighbours accounts (9, 2.1%) (Fig. 1 below).

### Knowledge of respondents about preparation for birth and its complication

Among 422 participants, 212 (50.2%) reported that they knew about identifying their place of birth. Out of all 422 respondents who knew about birth preparedness and its complication readiness, 289 (68.5%) reported as they expressed why to save money. The respondents who knew to identify skilled health care provider constitutes 119 (28.2%). Majority of them 253(60%) had reported as they knew about danger sign of obstetrics. Almost half 196 (46.4%) of the respondents mentioned arranging means of transportation for emergency. On the other hand, 68 (16.1%) of participants knew about the means of transportation for birth. Out of all 422 respondents, 129 (30.6%) of primigravida women expressed to arrange a way of communication to the source of help. It was only 48 (11.4%) respondents knew to arrange compatible blood donors (Table 7).

**Table 7:** Knowledge about birth preparedness and complication readiness among primigravida women in A.A Government Health facility, (*n* = 422) May, 2015

Variables	Response	Frequency	Percent
Identify place of delivery	Yes	212	50.2
	No	210	49.8
Saving money	Yes	289	68.5
	No	133	31.5
Identify skilled health care provider	Yes	119	28.2
	No	303	71.8
Means of transportation for emergency	Yes	196	46.4
	No	226	53.6
Arranging a way to communicate with a source of help	Yes	129	30.6
	No	293	69.4
Means of transportation for birth	Yes	68	16.1
	No	354	83.9
Awareness on danger sign of obstetrics	Yes	253	60.0
	No	169	40.0
Identify compatible blood donors	Yes	48	11.4
	No	374	88.6
(Others)^a^	Yes	20	4.7
	No	374	88.6

Mentioned more than one response

^a^to prepare porridge flour, baby cloths

### Well knowledgeable on birth preparedness and complication readiness

Of 355 primigravida women who had awareness, 113 (26.8%) were knowledgeable on danger sign during pregnancy. Among 302(71.6%) primigravida women 47 (11.1%) were knowledgeable on danger sign during delivery. Besides to this, out of 266 (63%) of respondents 60 (14.2%) were found being knowledgeable on identifying danger sign during postpartum period. Out of 277(65.6%) of the respondents 46 (10.9%) of the responds were knowledgeable about danger sign of new born. However, from 422 respondents it was only 64 (15.2%) who were knowledgeable about birth preparedness and complication readiness (Table 8).

**Table 8:** Respondents considered knowledgeable and well prepared for birth and its complication among primigravida women in A.A Government Health facility, May, 2015

Variables	Response	Frequency	Percent
Knowledgeable about danger sign during Pregnancy (*n* = 355)	Yes	113	26.8
	No	242	57.3
Knowledgeable about danger sign during labour and delivery (*n* = 302)	Yes	47	11.1
	No	255	60.2
Knowledgeable about danger sign during Postnatal (*n* = 266)	Yes	60	14.2
	No	206	48.6
Knowledgeable about danger sign of new born (*n* = 277)	Yes	46	10.9
	No	231	54.7
Knowledgeable about birth preparedness and complication readiness (*n* = 422)	Yes	64	15.2
	No	358	84.8

### Pregnancy related topics provided for primigravida women

From the total 422 respondents almost all 390 (92.4%) were provided with education about danger sign. There were also 363 (86.0%) who have learned about PMTCT. The respondents who got awareness about early and exclusive breast feeding were 394 (93.4%). While 220 (52.1%) of the respondents taught about sleeping under ITN and 265 (62.8%) of the respondents got lesson about parenting skills. Other topics were provided for more than two third of the respondents (Table 9).

**Table 9:** Respondents for provided pregnancy related topics among primigravida women in A. A. Government Health facilities, (*n*=422) May, 2015

Variables	Response	Number	Percent
Danger sign	Yes	390	92.4
	No	32	7.6
PMTCT	Yes	363	86.0
	No	59	14.0
Early and exclusive breast feeding	Yes	394	93.4
	No	28	6.6
Smoking cessation avoidance of alcohol, drugs and harm full practice	Yes	389	92.2
	No	33	7.8
Hygiene	Yes	340	80.6
	No	82	19.4
Sleep under ITN	Yes	220	52.1
	No	202	47.9
Family planning	Yes	377	89.3
	No	45	10.7
Birth and Complication readiness	Yes	349	82.7
	No	73	17.3
Next appointment time	Yes	408	96.7
	No	14	3.3
Parenting skills	Yes	265	62.8
	No	157	37.2

### Association of maternal socio-demographic variables with their knowledge of birth preparedness and complication readiness

In the bivariate and multivariable analysis significant association was observed between the marital status of mothers and their knowledge about birth preparedness and complication readiness. Married mothers were 11% less knowledgeable about birth preparedness and complication readiness than those who were unmarried [AOR = 0.110, 95% CI (0.026–0.461);]. Others maternal socio demographic variables did not have association (Table 10).

**Table 10:** Association of maternal socio-demographic variables with their knowledge on birth preparedness and complication readiness among primigravida women in A. A. Government Health facilities, (*n* = 422) May, 2015

Variable	Knowledge of birth preparedness and complication readiness				
	Category	Yes	No	COR (95% CI)	AOR (95% CI)
Age	15–20	5	41	1	
	21–26	31	158	1.609 (0.589,4.396)	
	27–32	26	132	1.615 (0.583,4.476)	
	33+	2	27	0.607 (0.110,3.359)	
	Total	64	358		
Marital status	Married	48	311	0.453 (0.238,0.863)*	0.110 (0.026–0.461)*
	Not Married	16	47	1	1
	Total	64	358		
Her educational status	Illiterate	10	50	1	
	Primary	19	96	0.990 (0.428,2.289)	
	Secondary and above	35	212	0.825 (0.383,1.778)	
	Total	64	358		
Her occupation	House wife	26	125	1	
	Gov’t. and Private	31	179	0.833 (0.471,1.471)	
	Employee	7	54	0.623 (0.255,1.523)	
	Non-employee	64	358		
	Total				

*(boldface) *p* value < 0.05

### Association of husband socio-demographic and maternal obstetrics factors with their knowledge on birth preparedness and complication readiness

In the bivariate and multivariable analysis significant association was observed between house hold monthly income and their knowledge about birth preparedness and complication readiness. Family monthly household income 1000–3000.00 birrs was found to be three times more knowledgeable about birth preparedness and complication readiness than who got more than 3000 birrs [AOR = 3.362(1.203, 9.393);]. But others husband socio demographic variables and maternal obstetrics factors did not associate with birth preparedness and complication readiness (Table 11).

**Table 11:** Association of husband socio-demographic and obstetrics factors with their knowledge on birth preparedness and complication readiness among primigravida women in A. A. Government Health facilities, May, 2015.cont’d

Variable	Knowledge of birth preparedness and complication readiness				
	Category	Yes	No	COR (95%CI)	AOR (95%CI)
Husband education (*N* = 368)	Illiterate	2	16	1	
	Primary	8	48	1.33 (0.256,6.940)	
	Secondary and above	38	256	1.187 (0.263,5.37)	
Husband Occupation (*n* = 368)	Gov’t. and Private employee	37	236	1.197 (0.58,2.454)	
	Nonemployee	11	84	1	
Household Income (*n* = 368)	< 1000.00 birr	10	44	1.963 (0.868,4.440)	1.040 (0.178,6.088)
	1000–3000.00 birr	32	124	2.229 (1.238,4.013)*	3.362 (1.203,9.393)*
	> 3000.00 birr	22	190	1	1
Start ANC (in month) (*n* = 422)	< 4	44	242	1.455 (0.323,6.549)	
	5–7	18	100	1.440 (0.305,6.807)	
	> 7	2	16	1	
Gestational Age (*n* = 422)	1–4	15	73	1	
	5–7	18	141	0.621 (0.296,1.304)	
	> 7	31	144	1.048 (0.532,2.063)	
No. of ANC visit (*n* = 422)	1	18	84	1	
	2–4	34	214	0.741 (0.397,1.385)	
	> 4	12	60	0.933 (0.419,2.081)	

*(boldface) *p* value < 0.05

### Association between maternal knowledge of danger signs and their knowledge on birth preparedness and complication readiness

On binary logistic regression, knowledge of danger signs during pregnancy, child birth/ labour, postnatal and neonatal period were found to have statistically significant association with birth preparedness and complication readiness.

Multiple logistic regression analysis was also computed to control the possible confounder, explores the association between independent variables, and birth preparedness and complication readiness. Knowledge of danger signs during child birth/ labour, postnatal period was significantly associated with birth preparedness and complication readiness. The adjusted odd ratio of birth preparedness and complication readiness was almost four times greater among knowledgeable for key danger sign of labour when compared to not knowledgeable. [AOR = 3.685, 95% CI (1.157, 11.737);]. Additionally, the adjusted odd ratio of birth preparedness and complication readiness among knowledgeable respondents about key danger signs during postpartum period were five times more than when compared to those who lack knowledge about it [AOR = 5.117,95% CI (1.388,18.863);] (Table 12).

**Table 12:** Association between maternal knowledge of danger signs and their knowledge on Birth preparedness and complication readiness among primigravida women in A. A. Government Health facilities, May, 2015.cont’d

Variable	Knowledge of birth preparedness and complication readiness			
	Category		COR (95%CI)	AOR (95%CI)
Knowledge status of danger signs during pregnancy (*n* = 355)	Not knowledgeable	242	1	1
	Knowledgeable	113	**3.003 (1.67,5.379)**	1.389 (0.517,3.729)
Knowledge status of danger signs during labour (*n* = 302)	Not knowledgeable	255	1	1
	Knowledgeable	47	**6.821 (3.387,13.733)**	3.685 (1.157,11.737)*
Knowledge status of danger signs during postpartum period (*n* = 266)	Not knowledgeable	206	1	1
	Knowledgeable	60	**6.561 (3.259,13.208)**	5.117 (1.388,18.863)*
Knowledge status of danger signs during neonatal period (*n* = 277)	Not knowledgeable	231	1	1
	Knowledgeable	46	**7.556 (3.738,15.271)**	1.694 (0.533,5.385)

*COR* Crud odds ratio, *AOR* Adjusted odds ratio

*(boldface) *p* value < 0.05

## Discussion

This study was conducted to assess the knowledge of birth preparedness and complication readiness among primigravida women in Addis Ababa governmental health facilities. Birth Preparedness and Complication Readiness (BP and CR) is a strategy to promote the timely use of skilled maternal and neonatal care, especially during childbirth, theoretically, preparing for childbirth and being ready for complications reduces delays in obtaining this care [7].

Knowledge of danger signs of obstetric complications during pregnancy, labour, postnatal and neonate period is the first essential step for appropriate and timely referral [13]. The findings of the study had provided an insight information on primigravida women’s knowledge about birth preparedness and complication readiness in the study area, which could help in designing appropriate interventions and as a base for further wide scale studies in the country.

In this study the proportion of respondents who were aware of danger signs of pregnancy were 355(84.1%); this is slightly higher compared to Mekelle town study [11]. Regarding knowledge, this study showed only 113(26.8%) of respondents were knowledgeable on danger signs during pregnancy. From those spontaneously mentioned knowledge of danger signs during pregnancy; only 319 (75.6%) mentioned vaginal bleeding, which is high compared to the study done in Malawi 62% [16], whereas very high when compared to studies in Adigrat town, North part of Ethiopia [3] and in Robe wereda, Arise zone [13]. The second and third key danger signs mentioned were swollen hands and face which is 216 (51.2%) and blurred vision 211 (50%), respectively. This is high comparing with the study done in Robe wereda, Aresi zone 2.2 and 37.7% [13]. In this study 168 (39.8%), 187(44.3%) and 113 (26.8%) spontaneously mentioned at least one, two and all three key danger signs of pregnancy, respectively. In contrary, 15.4% spontaneously mentioned at least one key danger sign, 2.6% mentioned at least two key danger signs and 0.4% mentioned all three key danger signs [3]. These differences might be due to geographical location; health facilities proximity, health facilities availability, parity and use of methodology.

According to this study, 302(71.6%) primigravida women have information on key danger signs during labor /child birth while only 47(11.1%) of respondents were found knowledgeable. Spontaneously mentioned danger signs during child birth were; vaginal bleeding followed by labor lasting more than 12 h, convulsion, and the placenta not delivered 30 min after the neonate. This finding was higher compared to the study of Uganda, Mulago hospital. In this study only 47(11.1%) spontaneously mentioned all four key danger signs of labor/ child birth while the study done in Nigeria showed high, which is 19.62% [17]. On the other side, a study in Adigrat town, North Ethiopia showed only 0.2% respondent named all four key danger signs [3]. This variation might be due to socio cultural, client awareness, study period and parity.

Regarding to knowledge of the primigravida women during post-partum period, in this study, 266 (63%) respondents have information, whereas, 60 (14.2%) of respondents were knowledgeable. Spontaneously mentioned danger signs during post-partum period were; vaginal bleeding 234 (55.5%), foul smelling vaginal discharge 132 (31.3%) and high fever 89 (21.1%). This result was relatively high compared to Uganda study [10]. In addition, in this study; study participants spontaneously mentioned all the three key danger signs were 60 (14.2%), which is relatively lower than the study done in Nigeria (21.73%) 17]. On the contrary, Adigrat town, North Ethiopia study; showed 0.4% mentioned all three key danger signs [3]. These result discrepancies might be due to parity, socio culture, access to information and time of maternal intervention. In this finding, the knowledge of danger signs during pregnancy, labor and postpartum, more than half of the respondent mentioned vaginal bleeding as a danger sign.

In this study among 277(65.6%) participants who reported that they have information about danger signs during neonatal period, only 40 (10.9%) were knowledgeable. The study revealed below half of participants 185 (43.8%) spontaneously mentioned difficulty or fast breathing as danger sign during neonatal period, this finding is low compared to the study done in Aleta wondo district, Sidama zone, Southern Ethiopia [18]. In addition, 50 (11.8%) of respondents spontaneously mentioned at least three key danger signs, which is three times more than the finding in Malawi study, [19]. 46 (10.9%) respondent spontaneously mentioned all four key danger signs of neonate which was almost similar with the study done in Nigeria, [17]. The similarity might be due to similar maternal and child health intervention. Generally, obstetrics danger signs are preventable and avoidable [10]. The respondents’ knowledge on obstetrics complications were low. The participants who were knowledgeable about obstetric complications during pregnancy better than in postnatal, in labour/ child birth and neonatal period which indicate the need of concerned effort in increasing the awareness and knowledge of pregnant mothers and particularly to the primigravida women.

Birth preparedness and complication readiness (BP/CR) is a relatively common strategy employed by numerous groups implementing safe motherhood programs [7].

From 442 primigravida women, 375 (88.8%) of the respondents speak out that they have ever heard the term birth preparedness. This finding was high compared to Basoliben district, Amhara Regional State, North West Ethiopia study [20]. The source of information to hear the term birth preparedness were a health profession, family, HEW, CHW, media and others, chronologically. In this study, among the primigravida women that have information, 64 (15.2%) reported four key birth preparedness and its complications domains which were “to identify place of delivery”,” to identify skilled birth attendant”, “means of transportation and saving money mentioned “. This finding is almost similar with Robe wereda 16.5% [13] and Goba woreda, Oromia region, Ethiopia 14.6% [2]. “To identifying place of delivery” is very important especially in our setup. The skilled care provider for attending birth could be very important if she planned to deliver at the health institution. This study showed half of the respondents revealed to identify place of delivery 212 (50.2%) which is almost similar with the finding of Arisi zone Robe wereda 50.8% [13]. In contrast to these results, the study of Adigrat town, Northern Ethiopia, is very low which was only 26.2% [3]. Even though the mothers identify place of delivery, it could be difficult to secure transport at the time of emergency. In this finding, 202 (47.9%) of respondents spontaneously mentioned means of transportation for emergency and child birth as key birth preparedness and its complications domains which is lower than study done in Robe wereda 69.7% [13] but higher than Adigrat study [38]. With regards to skilled birth attendants, the respondents cited about only 119(28.2%) which is lower than Robe wereda study 68.5% [13], however; higher than Adigrat town finding which was7.9% [3]. The difference of the result in this regard might be due to geographical, cultural, parity, used methodology and other infrastructures. However, it seems some of the respondents consider that anything which is done before child birth like preparing flour for porridge and prepare cloth for new born baby rather than recommended elements which have to be done as birth preparedness.

Generally, most of the respondents heard the term birth preparedness from health professionals and their families. According to this study finding, more than three fourth of the primigravida women had no comprehensive knowledge on birth preparedness and its complication, also all the primigravida women have no any past experience for birth preparedness and complication readiness. Therefore, they need to know at least key elements of BP and CR. These may facilitate early decision and arrival to health facilities if they have any obstetrics problems.

The multivariable logistic regression revealed that marital status, monthly house hold income, knowledge on key danger sign of labour/child birth and postnatal period had association with knowledge of birth preparedness and complication readiness. Married women were by 11% [AOR = 0.110(0.026–0.461)] less knowledgeable about birth preparedness and complication readiness compared to unmarried women. This finding is unlikely to Adigrat town; Northern Ethiopia [3], this might be due the difference in sample size. This study revealed that women who had house hold income of 1000–3000 Ethiopian birr were more than three times [AOR = 3.362(1.203, 9.393)] more knowledgeable about birth preparedness and complication readiness than women who had income of greater than 3000 thousand birrs. Respondents who were knowledgeable for key danger signs of labour/ child birth were found to be more knowledgeable [AOR = 3.685, 95% CI (1.157, 11.737)] compared to those who are not. This finding is similar with Robe Woreda study [13]. In addition, those having knowledge about key danger signs of postpartum period were five times more knowledgeable than those who do not have knowledge about it [AOR =5.117(1.388, 18.863);]. This result is in line with the study conducted elsewhere in Ethiopia [2].

### Strength and limitation of this study

#### Strength of the study

- ➢ Selection bias was minimised by using probability sampling method.
- ➢ The study was done on the first time specifically only the primigravida women

#### Limitation of the study

- ➢ The study only relied on quantitative approaches
- ➢ Since the data collectors were health professionals there might have professional bias.
- ➢ The study was conducted in the health facilities due to this it does not include those primigravida women who did not come to the health facilities.
- ➢ Lack of similar studies in our country and other countries to make comparative discussion on specify the primigravida women.

## Conclusion and recommendation

### Conclusion

Despite the awareness of majority of the respondents on birth preparedness and complication readiness they were not knowledgeable about birth preparedness and complication readiness. Only small numbers of primigravida women were knowledgeable for danger signs of pregnancy, labour/child birth, postpartum and neonatal period. The marital status, monthly households’ incomes, knowledge of key danger signs during labour, and post-partum period were independent predictors of birth preparedness and complication readiness. Accordingly, this, information given about danger sign and birth preparedness during ANC follow up was not comprehensive. Family health care providers, health facility, other partners, program level managers and policy makers take their responsibility and work together to improve the service and increase knowledge on birth preparedness and complication readiness.

### Recommendation

Based on the findings, the following areas were identified and specific recommendations were made in different levels.

#### Facility level

- ➢ Design and implement health promotion activities; awareness creation, Health education and distribution of IEC materials and follow up at individual, family and community level.
  - To improve knowledge of pregnant mother about danger signs and birth preparedness: strengthen the health education system; provide health education for pregnant mothers and community members.
  - Prepare queue card (mother and baby card), that have information about danger signs and birth preparedness, counsel and give the card to remained.
  - Health care providers, who are working in ANC and maternity, counsel every pregnant mother depending on FANC guideline.

#### For policy and program level

- ✓ Addis Ababa city administrative health bureau, each sub city; Empower health care workers; by designing on job training, regular supportive supervision, coaching and monitoring.

### For future research

- ✓ Promote researchers to do more qualitative and quantitative research at community and facility level.

## Data Availability

The dataset is available in the form of Epi Data version 3.1 and SPSS version 20 up on request of authors.

## Abbreviations

ANC: Antenatal care
BP: Birth preparedness
CHW: Community Health Worker
CR: Complication readiness
EDHS: Ethiopian Demographic and Health Survey
EmONC: Emergency Obstetric and Neonatal Care
FANC: Focused antenatal care
HC: Health Centre
HEW: Health Extension Worker
HIV: Human Immunodeficiency Virus
JHPIEGO: Johns Hopkins Program for International Education in Gynaecology and Obstetrics
MDG5: Millennium Development Goal for maternal health
MMR: Maternal Mortality Ratio
MOH: Federal Ministry of Health
SPSS: Statistical Package for Social Science
WHO: World Health Organization

## References

[1] Tura G (2009). Antenatal care service utilization and associated factors in Metekel zone, northwest Ethiopia. Ethiop J Health Sci.

[2] Markos D, Bogale D. Birth preparedness and complication readiness among women of child bearing age group in Goba woreda, Oromia region, Ethiopia. BMC Pregnancy Childbirth. 2014:1–9 http://www.biomedcentral.com/14712393/14/282. 10.1186/1471-2393-14-282 PMC4148918 25132227

[3] Hiluf M, Fantahun M. Birth preparedness and complication readiness among women in Adigrat town, North Ethiopia. Ethiop J Health Dev. 2008;22(1):14–20.

[4] Conrad P, Schmid G, Tientrebeogo J, Moses A, Kirenga S, Neuhann F, Muller O, Sarker M (2012). Compliance with focused antenatal care services: do health workers in rural Burkina Faso, Uganda and Tanzania perform all ANC procedures?. Trop Med Int Health.

[5] Health Sector Development Programme IV Annual Performance Report EFY 2003 (2010/2011). Federal Democratic Republic of Ethiopia, Ministry of Health, Addis Ababa.

[6] Anya SE, Hydaraand A, Jaiteh LES. Antenatal care in The Gambia: missed opportunity for information, education and communication. BMC Pregnancy Childbirth. 2008:1–7. 10.1186/1471-2393-8-9. 10.1186/1471-2393-8-9 PMC2322944 18325122

[7] JHPIEGO. Monitoring birth preparedness and complication readiness tool and indicator for maternal and new born care. Matern Neonatal Health Program. 2009:21231–3492 USA (www.jhpiego.org). Accessed 15 Jan 2015.

[8] Kushwah SS, Dubey DK (2008). A study for assessing birth preparedness and complication Readiness intervention in rewa district of madhya Pradesh.

[9] Smith PK (2012). Birth preparedness and complication readiness of aredetedsoial health activities under national health mission in Rural Karanataka, India.

[10] Mbalinda SN, Nakimuli A, Kakaire O, Osinde MO, Kakande N, Kaye DK. Does knowledge of danger signs of pregnancy predict birth preparedness? A Critique of the evidence from women admitted with pregnancy complications. Health Res Policy Syst. 2014:12–60. 10.1186/1478-4505. 10.1186/1478-4505-12-60 PMC4197291 25300499

[11] Weldearegay HG (2015). Determinant factors of male involvement in birth preparedness and complication readiness at Mekelle town; a community-based study. Sci J Public Health.

[12] Federal Democratic Republic of Ethiopia Ministry of Health. Maternal Death Surveillance and Response (MDSR) technical guideline. Addis Ababa: Federal Democratic Republic of Ethiopia Ministry of Health; 2012.

[13] Kaso M, Addisse M. Birth preparedness and complication readiness in Robe Woreda, Arsi Zone, Oromia Region, Central Ethiopia: a cross-sectional study. Reprod Health. 2014:1–12. 10.1186/1742-4755-11-55. 10.1186/1742-4755-11-55 PMC4118259 25038820

[14] Gebrehiwot H, Bahta S, Haile N. Awareness of danger signs of pregnancy and its associated factors among pregnant women who visit ANC in Mekelle public hospitals. Am J Adva Drug Deliv. ISSN 2321-547X (www.ajadd.co.uk).

[15] Central Statistical Agency and ORC Macro (2006). Ethiopian demographic and health survey 2005. Addis Ababa, Ethiopia and Calverton, Maryland, USA.

[16] Kumbani LC, Mclnerney P (2006). Primigravida knowledge about obstetric complications in an urban health centre in Malawi. Curationis.

[17] Emma-Ukaegbu UC, Nwokeukwu HI, Uzochukwu BSC (2014). An assessment of birth preparedness and complication readiness in antenatal women in Umuahia north local government area, Abia State, Nigeria. IOSR J Dent Med Sci.

[18] Hailu M, Gebremariam A, Alemseged F. Knowledge about obstetric danger signs among pregnant women in aleta wondo district, sidama zone, Southern Ethiopia. Ethiop J Health Sci. 2010;20(1). 10.4314/ejhs.v20i1.69428 PMC3275898 22434957

[19] Botha AK, Maluwa A, Pindani M, Bultemeier K (2013). Birth preparedness and complication readiness among postnatal mothers in Malawi. Health.

[20] Bishaw W, Awoke W, Teshome M (2014). Birth preparedness and complication readiness and associated factors among pregnant women in Basoliben District, Amhara Regional State, Northwest Ethiopia, 2013. Prim Health Care.

